# The Impact of COVID-19 on Depressive Symptoms through the Lens of Sexual Orientation

**DOI:** 10.3390/brainsci11040523

**Published:** 2021-04-20

**Authors:** Mariana Duarte, Henrique Pereira

**Affiliations:** 1 Department of Psychology and Education, Faculty of Social and Human Sciences, University of Beira Interior, Pólo IV, 6200-209 Covilhã, Portugal; mariana.r.duarte@ubi.pt; 2 Research Centre in Sports Sciences, Health Sciences and Human Development (CIDESD), 5001-801 Vila Real, Portugal

**Keywords:** depressive symptoms, fear of COVID-19, negative impact of COVID-19, sexual orientation, LGB people

## Abstract

This research seeks to explore the impact of COVID-19 on depressive symptoms, analyzing discrepancies of sexual orientation in a Portuguese-speaking sample. 1590 individuals participated, of which 63% were women, and 88% self-identified as straight. Participants responded to the depression sub-scale of the Beck Symptoms Iventory-18, the fear of COVID-19 scale and the COVID-19 negative impact scale. Depressive symptoms observed were higher than expected, and several significant differences were obtained: women and self-identified bisexual participants had higher levels of depressive symptoms compared to male and straight and gay or lesbian participants. Depressive symptoms negatively correlated with age and positively correlated with COVID-19 aggravated responses, fear of COVID-19, and negative impact of COVID-19. Hierarchical linear regression analysis showed that age, gender and sexual orientation explained 6% of the variance of depressive symptoms, and when fear and the negative impact of COVID-19 was added, the model explained 23% of results. This study provides an important contribution to the understanding of factors arising from the pandemic that may have an impact on the mental health of sexual minorities.

## 1. Introduction

Corona Virus Disease 2019 (COVID-19), caused by the SARS-CoV-2 virus, manifests itself as a severe acute respiratory syndrome [1]. The first case was diagnosed in China in December 2019, spreading to different locations and populations around the world [2,3]. Due to its rapid spread and the increase in the number of critically ill patients, in just a few months, the World Health Organization (WHO) declared a state of pandemic worldwide [4]. As of early March 2021, the SARS-CoV-2 virus had infected approximately 115,660,000 people worldwide, and nearly 2,572,000 died from complications of the disease [5].

Since it was declared a pandemic, the spread of COVID-19 has been the focus of attention for governments and populations [6], having turned into a crisis at various levels with consequences worldwide [7]. One of the main concerns is the impact of this pandemic on the mental health of the population [6]. Several studies that investigated mental health showed a higher prevalence of depression, anxiety and insomnia in the population during the outbreak of COVID-19, concluding that the pandemic had a negative impact on the mental health of the population [8,9,10]. In a study carried out in Brazil, almost half of the samples showed sadness and symptoms of depression during the pandemic [11]. In Portugal, several investigations have shown that participants revealed significantly high levels of depression [12] and sadness during lockdown [13].

In addition to the psychological implications related to COVID-19, measures implemented to contain the disease may also consist of risk factors for mental health [6]. Social distancing and other measures that suspend day-to-day activities, despite protecting the physical health of the population, are associated with the development of various disorders, such as depression [4,14,15,16]. In this sense, negative effects were identified, such as symptoms of post-traumatic stress, confusion and anger at the measures implemented [17,18]. It was also concluded that people who were quarantined showed a higher prevalence of presenting depression compared to those who were not [11]. Exposure to news related to COVID-19, misinformation about the virus and consumption of false news, are also risk factors that can lead to increased fear, anxiety and feelings of sadness [19].

Lesbian, gay and bisexual (LGB) people are one of the groups affected by COVID-19 [20], as these individuals were already at a disadvantage due to the stigma of society against their sexual orientation [21]. According to the minority stress theory, stigma, prejudice and discrimination against LGB people generates stress, ultimately having a negative impact on their mental health [22]. During the pandemic it was found that LGB individuals had less contact with their community, which would be essential for reduced psychological suffering [23]. These minorities may also have faced family conflicts, as they may have had the need to stay at home longer [9], and complications in accessing HIV prevention and treatment [24]. Such conditions present challenges to their mental health, including increased symptoms of depression [25].

Homosexuality was considered a mental disorder until 1973 by the Diagnostic and Statistical Manual of Mental Disorders (DSM) [26]. However, even after this classification was withdrawn, several discussions arose mainly within the religious community with a homophobic stance, allowing this “label” to exist until present day [27,28]. Generally, LGB people have a higher prevalence of mental health problems [29], including high levels of depression and substance use [30] compared to their straight peers [31], and bisexual people could be more at risk [32]. Among sexual minorities, there are indications that bisexual people have higher levels of suicidal ideation [33] than homosexual individuals [34]. Bisexual people consider that they are less connected to the LGBT+ community, as they face discrimination and exclusion within the community itself [35].

In addition, the LGB population is subject to a stressful social environment created from stigma, prejudice, social exclusion, hatred and violence, often presenting a feeling of shame about their sexuality [36], with these phenomena being observable in cultures such as in Portugal and Brazil. Indeed, Brazil is one of the countries with the highest rates of violence against the LGB population [37], triggered by homophobia and prejudice [38]. The heteronormativity model imposed by society makes the LGB population more vulnerable to discrimination, intolerance and attitudes about hate heterosexist individuals [39]. In Portugal, despite the existence of a non-discrimination clause based on sexual orientation in the Constitution, a law that allows same-sex couples to marry (since 2011), and a law that allows same-sex couples to adopt and joint-adopt children (since 2016), discriminatory practices continue to exist within the population [40,41,42]. Thus, discrimination and violation of the rights of the LGB population, mainly due to moral grounds and religious conservatism [29], generates intense suffering, anguish and insecurity, leaving this minority more likely to manifest depressive symptoms [35].

Some investigations have revealed the negative impact of the pandemic on the mental health of LGB individuals [19,43,44]. In this sense, Barrientos et al. [45] reported that, in their study, LGB participants suffered a huge negative psychosocial impact due to the COVID-19 pandemic, and Moore et al. [46] stated that sexual and gender minorities had higher levels of depression. However, given that there are no studies carried out on Portuguese-speaking populations, the present study was developed to assess the impact of COVID-19 on the levels of depressive symptoms through the lens sexual orientation.

## 2. Materials and Methods

*Sample Size.* The Portuguese and Brazilian population size combined equals around 220 million. We used a sample size calculator to compute the minimum number of necessary samples to meet the desired statistical constraints, applying the following formula: n = N × X/(X + N − 1), where Z_α/2_^2^ × *p* × (1 − *p*)/MOE^2^, and Z_α/2_ is the critical value of the normal distribution at α/2 (for a confidence level of 99%), MOE is the margin of error, *p* is the sample proportion, and N is the population size. The result was 666 participants to have a confidence level of 99% that the real value was within ±5% of the surveyed value. Nevertheless, we were able to reach a number of participants well above that number (*n* = 1590).

*Procedures.* This research was carried out through an online website that was available between October and December 2020. This link, carried out through Microsoft Forms, was disseminated through social networks and e-mail addresses. Participation was voluntary, and participants were referred to a linked website created specifically for the purpose of this investigation. The first page of the questionnaire explained the objectives of the study, and informed participants about how to fill it in, how to withdraw from the study, and how to contact the authors for more information. They were also asked to read and agree to an informed consent waiver.

A total of about 8000 notifications were sent, and 1667 participants responded voluntarily (21% response rate), however 77 participants failed to meet the inclusion criteria, and were eliminated. This notwithstanding, 1590 participants fully met the inclusion criteria (20% response rate). The dissemination of the survey complied to all of the ethical principles of informed consent, anonymity and confidentiality. Neither rewards nor other incentives were offered. Inclusion criteria included the following: being older than 18 years of age, to be a Portuguese native speaker (from Portugal or Brazil).

*Measurements.* The research protocol used in the present research encompasses four instrument measures: the sociodemographic questionnaire, the Brief Symptom Inventory 18 (BSI-18) and the scales of fear in relation to COVID-19 and the negative impact in relation to COVID-19.

*Demographic Information*. The sociodemographic information of the sample participants was collected through a questionnaire. Participants were asked about their age, gender, nationality, sexual orientation, marital status, socioeconomic status, among other characteristics.

*Depressive Symptoms*. Participants responded to the depressive symptoms sub-scale of the Portuguese version of the Brief Symptom Inventory 18 (BSI-18) [47] in order to assess the possible problems or symptoms experienced in the previous week. The depression subscale encompasses six items focusing on the main symptoms of depressive disorders (e.g., feeling blue, feeling no interest in things, feeling lonely, feeling hopeless about the future, feeling worthless, and having suicidal thoughts). Likert-type responses assessing frequency of symptoms varied between 0-Never and 4-Always. The global severity index, provides a measure of the individuals’ levels of psychological malaise is obtained from the mean of the six items on the scale, with the highest scores revealing a more intense psychosymptomatology. The internal consistency obtained in the present study was α = 0.94, which reveals excellent reliability. To complement the assessment of depression symptoms associated with the COVID-19 pandemic, a question was asked regarding the participants’ perception of the worsening responses on the depression subscale due to the pandemic: “how were the responses you gave to the previous questions increased by the COVID-19 pandemic?” Likert-type responses varied between 1-Nothing and 6-A lot.

*Fear of and Negative Impact of COVID-19*. Participants also responded to the fear of COVID-19 scale and the negative impact of COVID-19 scale. The fear of COVID-19 scale was originally developed by Ahorsu et al. [48], and as in the original version, the Portuguese version comprised seven items, ranging in score from 1 to 5 as measured by a Liker-type scale, with higher scores indicating a greater the fear of COVID-19 [49]. Examples of questions are as follows: “It makes me uncomfortable to think about Corona”, “When I watch news and stories about Corona on social media, I become nervous or anxious” or “I am afraid of losing my life because of Corona”. Regarding the negative impact of COVID-19 scale, it allowed measuring the participants’ perception of the negative impact that the pandemic had on their lives. It consisted of ten items related to the various areas of psychosocial functioning, ranging in score from 1 to 5 as measured by a Liker-type scale with higher scores meaning greater negative impact of COVID-19 [49]. Examples of questions are as follows: “Compared to my life before the COVID-19 pandemic, it had a negative impact ... on my professional or academic life, …on my family life, …on my financial life, etc.” The internal consistency obtained was α = 0.87 for both scales, which indicates excellent reliability.

*Data Analysis*. Descriptive statistics were performed to describe the sample (mean, standard deviation, frequencies and percentages). Student *t*-tests, and one-way ANOVAs were conducted to evaluate differences between comparison groups. To assess the association between relationship fear of COVID-19, negative impact of COVID-19 and depressive symptoms, Pearson correlation coefficients were conducted. Finally, A hierarchical linear regression analysis was conducted to examine the effects of independent variables (age, gender, sexual orientation, fear of COVID-19, and negative impact of COVID-19) on the dependent variable (depressive symptoms).

## 3. Results

A total of 1590 participants (63.0% women, 37.0% men) aged between 18 and 74 years took part in the study (*M_age_* = 33.67; *SD* = 12.95). Regarding nationality, 76.8% were Portuguese and 23.2% Brazilian. The majority of participants (56.8%) reported being single, holding a university degree (68.8), living in small urban environments (42.2%), and belonging to a middle socioeconomic status (57.8%). Regarding sexual orientation, 1399 individuals (88.0%) identified as straight, while 97 participants (6.1%) identified as bisexual, and 94 participants (5.9%) identified as gay or lesbian. Regarding professional status, 49.0% of the participants claimed to be employed. The sample’s sociodemographic data are shown in Table 1 in greater detail.

Mean scores for depressive symptoms were *M* = 1.01 (*SD* = 0.86), which was slightly above what would be expected when compared to the ommunity sample (without psychopathology) drawn from literature (*M* = 0.89, *SD* = 0.72) [50]. The independent samples *t*-test demonstrated the existence of statistically significant differences in depressive symptoms by genders (*t*(1335) = 4.108; *p* < 0.001), indicating that female participants had higher levels of depressive symptoms (*M* = 1.08; *SD* = 0.85) than male participants (*M* = 0.88; *SD* = 0.85) (see Table 2).

One-way analysis of variance (ANOVA) showed statistically significant differences in depressive symptoms by sexual orientation (*F*(20.257; 0.701) = 27.809; *p* < 0.001), indicating that self-identified bisexual participants scored higher (Table 2). The post-hoc test (Tukey) confirmed the statistically significant differences between bisexual and straight participants (*p* < 0.001), between bisexual and gay and lesbian participants (*p* = 0.002), and between straight, and gay and bisexual participants (*p* < 0.05) (Table 3).

Mean scores for fear of COVID-19 were *M* = 2.44 (*SD* = 0.84). The independent samples *t*-test demonstrated the existence of statistically significant differences in fear of COVID-19 by gender (*t*(1418) = 6.876; *p* < 0.001), indicating that female participants had higher levels of fear of COVID-19 (*M* = 2.55; *SD* = 0.83) than male participants (*M* = 2.23; *SD* = 0.82) (see Table 4).

One-way analysis of variance (ANOVA) showed statistically significant differences in fear of COVID-19 by sexual orientation (*F*(4.258; 0.708) = 6.014; *p* = 0.003), indicating that self-identified bisexual participants scored higher. The post-hoc test (Tukey) confirmed the statistically significant differences between bisexual and straight participants (*p* = 0.006) (see Table 5).

Mean scores for the negative impact of COVID-19 were *M* = 2.60 (*SD* = 0.88). The independent samples *t*-test demonstrated no statistically significant differences by gender (*t*(1408) = 1.232; *p* = 0.186) (see Table 6).

One-way analysis of variance (ANOVA) showed statistically significant differences in the negative impact of COVID-19 by sexual orientation (*F*(14.697; 0.762) = 19.282; *p* < 0.001), indicating that self-identified bisexual, and gay or lesbian participants scored higher. The post-hoc test (Tukey) confirmed the statistically significant differences between straight and bisexual participants, and between straight and gay or lesbian participants (*p* < 0.001) (see Table 7).

Pearson’s correlation coefficient test showed that depressive symptoms correlated negatively with age (*r* = −0.156; *p* < 0.001), positively with the perception of how COVID-19 aggravated responses to the Depression Symptoms Scale (*r* = 0.441; *p* < 0.001), positively with the fear of COVID-19 (*r* = 0.257; *p* < 0.001), finally, also positively with negative impact of COVID-19 (*r* = 0.421; *p* < 0.001) (see Table 8).

We also carried out a hierarchical linear regression analysis to assess the effects of age, gender, sexual orientation, fear and negative impact of COVID-19 on the depressive symptoms of the sample. The variables “age”, “gender” and “sexual orientation” were added in the first block (Model I). COVID-19’s Fear and Negative Impact were added in the second block (Model II). The first block of analysis explained 6% of the variance of depressive symptoms, while the second block explained 23%. Thus, as shown in Table 9, all variables are strong predictors of depressive symptoms.

## 4. Discussion

This investigation sought to explore the impact of COVID-19 on depressive symptoms through the lens of sexual orientation in a Portuguese-speaking sample. Results showed that depressive symptoms are present in the study sample, being above what would be expected for a non-clinical community normative sample. This result is associated with the emergence of the COVID-19 pandemic. As found in other studies conducted in Portuguese-speaking samples [51], the majority of participants felt depressed during the period of national state of emergency due to changes in routines and decreased contact with friends and family caused by measures of social distance. In addition, the fear of infecting or being infected by COVID-19 [52], concerns about the professional future and financial instability [53] generated emotional vulnerability associated with depressive symptoms, especially among women [54,55,56]. This can be explained by the fact that women have a greater psychological vulnerability [57], poor adaptive coping styles, greater prevalence of somatic diseases and greater social and cultural insecurity [58]. Women also tend to ruminate more on negative thoughts, which prolongs their suffering [59]. In addition, the vision of women in society and the different roles played by women, such as carrying out domestic chores, providing care for children/the elderly and professional responsibilities, which were carried out simultaneously during the quarantine period [60], as well as the increase in domestic violence during this period [61] may correspond with the explanation of our results.

Participants who self-identified as bisexual also had higher levels of depressive symptoms as compared to participants who self-identified as straight, and gay or lesbian. These results are congruent with other studies that emphasize that the levels of depression among LGB individuals tend to be high [62], with a prevalence of two to four times higher compared to straight people [63]. Bostwick et al. [35] claim that it is bisexual individuals who have higher levels of mental health problems when compared to homosexual individuals, because bisexual individuals tend to hide their sexual orientation in different social contexts in order to protect themselves from discrimination and stigma [40]. Generally, bisexual individuals experience high levels of the three main stressors that make up the minority stress model—internalized homophobia, stigma and discrimination [22]. In addition, due to the COVID-19 pandemic and the measures adopted to contain it, individuals from sexual minorities demonstrated health problems and impaired well-being, which was reflected in the onset or worsening of depression symptoms during the first few months of the pandemic [46,64,65,66,67,68].

Our results also showed that older participants revealed fewer depressive symptoms. This confirms other research [69] reporting that younger people have higher levels of depression. However, most studies claim that stressful life events, such as the COVID-19 pandemic, are risk factors for depression in older people and that depressive symptoms increase with age [70]. The result of the present study seems to demonstrate that the ability to develop strategies and to adapt emotionally and psychologically to losses increases with age, which can decrease the risk factors for depressive symptoms [71]. In this sense, the fact that older adults are more effective in regulating their emotions when compared to younger adults helps to explain why depressive symptoms decrease with advancing in age [72].

The results also show that the COVID-19 pandemic appears to have aggravated the depressive symptoms of the sample participants. This result is in line with the existing literature [73], which demonstrated that when comparing the levels of depression before and during the pandemic among people without previous mental disorders or with less severe mental disorders, an increase in the levels of depressive symptoms was observed. We can see that the COVID-19 pandemic has had consequences for the entire population, having a negative impact in several areas. This impact is directly related to the fear of being infected, of infecting others or of dying from the virus [74], and also to the fear of losing one’s job or being socially excluded by friends and family [75].

Depressive symptoms increase according to the fear of COVID-19 felt by individuals and negative impact experiences associated with the pandemic. In fact, fear related to COVID-19 affects people’s psychological well-being and can lead to symptoms of depression [8], and overall negative psychological responses [76]. The measures adopted by governments to contain the pandemic, such as quarantine or social isolation, may have contributed to this psychological suffering [77]. This impact can also be reflected in the economic instability of the population, since the pandemic has left a large number of people unemployed [78]. This further intensifies the negative emotions experienced by individuals during the pandemic, which can lead to the development of depressive symptoms [79].

The comparison between the various dimensions that acted as predictors of depressive symptoms showed that age, gender, and sexual orientation, but mostly fear and the negative impact of COVID-19 contributed to the explanation of the depressive symptoms. The fact that the pandemic has had an impact on the health system, politics, the economy and education can be reflected in the appearance of several mental disorders, namely depression [16]. Women and individuals belonging to sexual minorities may have seen greater negative impact from COVID-19 and had higher levels of depression [52] and this level of concern and fear can be highly disabling [6,8,80].

This research is not without limitations. Firstly, this was a convenience sample collected online. Despite the fact that the proportion of LGB participants in the sample is reflective of the prevalence in population (around 10%), comparative analysis may have been subject to bias since comparison groups are disproportionate. Thus, these results cannot be generalized, although we believe that this is a credible option to promote the representation of sexual minorities in this study. Secondly, the sample was disproportionately differentiated, with contributions from two distinct cultural settings (Portugal and Brazil). Thirdly, as presented in the research, other variables can be predictors of depressive symptoms. In fact, depression can be related to factors such as loneliness [52,55], socioeconomic status [24,64,65,66] or maladaptive coping mechanisms [59], mainly among women [67,68]. Future studies should include more varied samples and other research methodologies, such as face to face inquiries or qualitative analysis. In addition, longitudinal methodologies would be important to assess the long-term impact of the COVID-19 pandemic on depressive symptoms in sexual minority groups.

## 5. Conclusions

This was an important contribution to the analysis of how the COVID-19 pandemic has unevenly affected the mental health of the world population. Those who were in a vulnerable situation suffered most from the consequences of this new reality, and this negative impact highlights the need for further research in the area of mental health, specifically with groups of greater vulnerability. Given that the consequences of this pandemic can prolong over time, it will be decisive to devise psychological intervention strategies in order to respond to the needs and difficulties of the groups most at risk such as women and LGB people. This study provides an important contribution to the understanding and clarification of factors arising from the pandemic that may have an impact on the mental health of sexual minorities.

## Figures and Tables

**Table 1:** Sociodemographic characteristics of the participants (*N* = 1590; *M_age_* = 33.68; *SD* = 12.95).

Variable	Category	*N*	*%*
Gender	Women	1002	63.0
	Men	588	37.0
Nationality	Portuguese	1221	76.8
	Brazilian	369	23.2
Marital Status	Single	903	56.8
	Married	412	25.9
	De facto union	167	10.5
	Divorced/Separated	94	5.9
	Widower	14	0.9
Educational Attainment	No schooling	2	0.1
	Up to 9 years of school	57	3.6
	Up to 12 years of school	437	27.5
	Undergraduate degree	537	33.8
	Postgraduate degree	388	24.4
	Ph.D.	169	10.6
Place of residence	Small rural	280	17.6
	Big rural	154	9.7
	Small urban	671	42.2
	Big urban	485	30.5
Socioeconomic Status	Low	68	4.3
	Middle-Low	383	24.1
	Middle	919	57.8
	Middle-High	205	12.9
	High	15	1.0
Sexual Orientation	Straight	1399	88.0
	Bisexual	97	6.1
	Gay or Lesbian	94	5.9
Professional Status	Unemployed	36	2.3
	Student	425	26.7
	Working student	186	11.7
	Self-employed	139	8.7
	Employed	779	49.0
	Retired	25	1.6

**Table 2:** Differences in Depressive Symptoms by Gender and Sexual Orientation.

Dependent Variable	Categories	Sub-Categories	*N*	*M*	*SD*	*t/F (df)*	*p*
Depressive Symptoms	Gender	Women	1002	1.08	0.86	4.108 (1335)	0.000 *
		Men	588	0.89	0.85		
	Sexual Orientation	Straight	1399	0.95	0.83	27.809 (20.257;0.701)	0.000 *
		Bisexual	97	1.61	0.97		
		Gay or Lesbian	94	1.17	0.90		

* *p* < 0.001.

**Table 3:** Results for Depressive Symptoms by comparison groups (Sexual Orientation).

Dependent Variable	(I) Sexual Orientation	(J) Sexual Orientation	Mean Difference I–J	*p*
Depressive Symptoms	Straight	Bisexual	−0.66075	0.000 **
		Gay or Lesbian	−0.22505	0.042 *
	Bisexual	Straight	0.66075	0.000 **
		Gay or Lesbian	0.43570	0.002 *
	Gay or Lesbian	Straight	0.22505	0.042 *
		Bisexual	−0.43570	0.002 *

* *p* < 0.05; ** *p* < 0.001.

**Table 4:** Differences in Fear of COVID-19 by Gender and Sexual Orientations.

Dependent Variable	Categories	Sub-Categories	*N*	*M*	*SD*	*t/F (df)*	*p*
Fear of COVID-19	Gender	Women	1002	2.55	0.83	6.876 (1418)	0.000 **
		Men	588	2.23	0.82		
	Sexual Orientation	Straight	1399	2.42	0.83	6.014 (4.258; 0.708)	0.003 *
		Bisexual	97	2.71	0.83		
		Gay or Lesbian	94	2.58	0.94		

* *p* < 0.05; ** *p* < 0.001.

**Table 5:** Results for Fear of COVID-19 by comparison groups (Sexual Orientation).

Dependent Variable	(I) Sexual Orientation	(J) Sexual Orientation	Mean Difference I–J	*p*
Fear of COVID-19	Straight	Bisexual	−0.29104	0.006 *
		Gay or Lesbian	−0.16757	0.161
	Bisexual	Straight	0.29104	0.006 *
		Gay or Lesbian	0.12347	0.600
	Gay or Lesbian	Straight	0.16757	0.161
		Bisexual	−0.12347	0.600

* *p* < 0.05.

**Table 6:** Differences in the Negative Impact of COVID-19 by Gender and Sexual Orientations.

Dependent Variable	Categories	Sub-Categories	*N*	*M*	*SD*	*t/F (df)*	*p*
Fear of COVID-19	Gender	Women	1002	2.63	0.87	1.232 (1408)	0.186
		Men	588	2.57	0.91		
	Sexual Orientation	Straight	1399	2.55	0.88	19.282 (14.697; 0.762)	0.000 *
		Bisexual	97	3.01	0.94		
		Gay or Lesbian	94	2.98	0.98		

* *p* < 0.001.

**Table 7:** Results for the Negative Impact of COVID-19 by comparison groups (Sexual Orientation).

Dependent Variable	(I) Sexual Orientation	(J) Sexual Orientation	Mean Difference I–J	*p*
Fear of COVID-19	Straight	Bisexual	−0.45154 *	0.000 *
		Gay or Lesbian	−0.42625 *	0.000 *
	Bisexual	Straight	0.45154 *	0.000 *
		Gay or Lesbian	0.02530	0.980
	Gay or Lesbian	Straight	0.42625 *	0.000 *
		Bisexual	−0.02530	0.980

* *p* < 0.001.

**Table 8:** Correlation Matrix.

Variables	1	2	3	4	5
1—Age	-				
2—COVID-19 Aggravated Responses	−0.085 **	-			
3—Fear of COVID-19	0.008	0.464 **	-		
4—Negative Impact of COVID-19	−0.037	0.557 **	0.402 **	-	
5—Depressive Symptoms	−0.156 **	0.441 **	0.257 **	0.421 **	-

** *p* < 0.001.

**Table 9:** Hierarchical linear regression analysis predicting Depressive Symptoms.

Variable	Model I			Model II		
	B	SE B	β	B	SE B	β
Age	−0.009	0.002	−0.140 **	−0.009	0.002	−0.139 **
Gender	−0.217	0.049	−0.123 **	−0.147	0.045	−0.084 *
Sexual Orientation	0.270	0.045	0.165 **	0.150	0.042	0.092 **
Fear of COVID-19				0.101	0.028	0.098 **
Negative Impact of COVID-19				0.359	0.026	0.371 **
*R* ^2^			0.060			0.230
F			27.361 **			76.727 **

* *p* < 0.05; ** *p* < 0.001.

## Data Availability

The data presented in this study are available upon request.
